# DNA Polymerase θ: A Unique Multifunctional End-Joining Machine

**DOI:** 10.3390/genes7090067

**Published:** 2016-09-21

**Authors:** Samuel J. Black, Ekaterina Kashkina, Tatiana Kent, Richard T. Pomerantz

**Affiliations:** Fels Institute for Cancer Research, Department of Medical Genetics and Molecular Biochemistry, Temple University Lewis Katz School of Medicine, Philadelphia, PA 19140, USA; Samuel.J.Black@temple.edu (S.J.B); tug40626@temple.edu (E.K.); kentta@temple.edu (T.K.)

**Keywords:** DNA polymerase, DNA repair, microhomology-mediated end-joining, alternative end-joining, replication repair, genome instability, cancer

## Abstract

The gene encoding DNA polymerase θ (Polθ) was discovered over ten years ago as having a role in suppressing genome instability in mammalian cells. Studies have now clearly documented an essential function for this unique A-family polymerase in the double-strand break (DSB) repair pathway alternative end-joining (alt-EJ), also known as microhomology-mediated end-joining (MMEJ), in metazoans. Biochemical and cellular studies show that Polθ exhibits a unique ability to perform alt-EJ and during this process the polymerase generates insertion mutations due to its robust terminal transferase activity which involves template-dependent and independent modes of DNA synthesis. Intriguingly, the *POLQ* gene also encodes for a conserved superfamily 2 Hel308-type ATP-dependent helicase domain which likely assists in alt-EJ and was reported to suppress homologous recombination (HR) via its anti-recombinase activity. Here, we review our current knowledge of Polθ-mediated end-joining, the specific activities of the polymerase and helicase domains, and put into perspective how this multifunctional enzyme promotes alt-EJ repair of DSBs formed during S and G2 cell cycle phases.

## 1. Introduction

**Figure 1:**
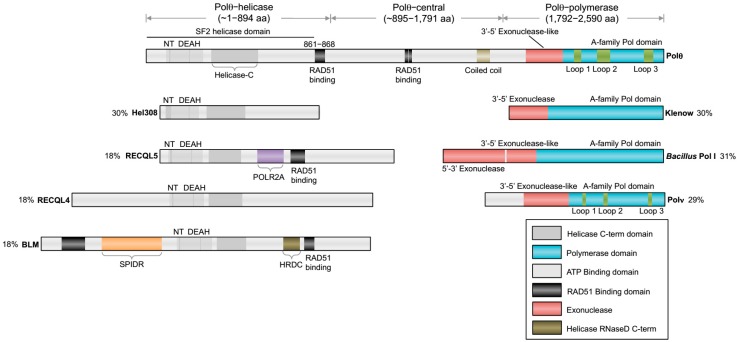
Schematic representation of Polθ and homologous proteins. The helicase, central and polymerase domains of *POLQ* are depicted at the top. The superfamily 2 (SF2) helicase domain contains a conserved nucleotide binding site (NT, dark grey), a conserved DEAH box motif (DEAH, dark grey), and a conserved helicase C-terminal domain (Helicase-C, dark grey). RAD51 binding domains (black) are found in the helicase and central domain. The polymerase domain contains a conserved A-family polymerase subdomain (blue), an inactive 3’–5’ exonuclease-like subdomain (red), and three unique insertion loops (green). Select *POLQ* polymerase and helicase orthologs are illustrated with their respective sequence similarities to *POLQ*. Relative homolog positions are based on sequence alignments. Characteristic regions found in helicase homologs are a RNA polymerase II subunit A binding subdomain (POLR2A, purple), a scaffolding protein involved in DNA repair (SPIDR, orange), and a helicase RNaseD C-terminal domain (HRDC, brown). Percent sequence similarities between homologous proteins and either Polθ-polymerase or Polθ-helicase domains are indicated. Sequence alignments and similarities were determined using Clustal Omega.

The *chaos1* (chromosome aberration occurring spontaneously 1) mutation in mice was discovered over 10 years ago as promoting a cellular phenotype, characterized by a high frequency of spontaneous and radiation, induced micronuclei in circulating red blood cells which are caused by defects in DNA repair or cell division [1]. Given that this particular mutation results in a major amino acid change from serine to proline in the *Polq1* gene, this seminal discovery stimulated a new line of research aimed at elucidating the function of this previously uncharacterized gene product referred to as Polθ which includes a C-terminal A-family DNA polymerase and an N-terminal superfamily 2 (SF2) Hel308-type DNA helicase (Figure 1). Following this initial discovery, the Wood lab purified full-length human Polθ and confirmed that it exhibits DNA synthesis and DNA-dependent ATPase activities [2]. Further studies in mice demonstrated that *Polq* functions in an ATM (ataxia telangiectasia mutated) independent manner and that *Polq^−/−^* *Atm^−/−^* double deficient cells are semi-synthetic lethal [3]. Subsequent research in mammalian systems showed that *POLQ* expression promotes cellular resistance to ionizing radiation which causes DNA double-strand breaks (DSBs) [4,5].

Later studies in *Drosophila* demonstrated that the *POLQ/Polq* ortholog *mus308* functions independently of the major DSB repair pathways non-homologous end-joining (NHEJ) and homologous recombination (HR), and that that cells mutated in *mus308* and *spn-A*, a *RAD51* ortholog, are hyper-sensitive to ionizing radiation [6]. These seminal studies in Drosophila also demonstrated that mus308 promoted the distinct DNA repair signature associated with alt-EJ which is characterized by relatively large deletions and insertions (indels) as well as the presence of microhomology flanking a significant fraction of repair junctions [6]. Taken together, these early studies indicated an important function for Polθ in alt-EJ repair of DSBs.

Prior studies in invertebrates showed that *Polq-1/mus308* promotes resistance to genotoxic agents that cause interstrand crosslinks in DNA that are normally repaired by a large ensemble of DNA repair enzymes from multiple pathways including HR, translesion synthesis and nucleotide excision repair [7,8,9,10]. A role for mammalian Polθ in interstrand crosslink (ICL) repair, however, has yet to be identified. Thus, the molecular basis for the apparent differential role for Polθ involvement in ICL repair between invertebrates and vertebrates remains unclear.

Intriguingly, several lines of evidence suggest that mammalian Polθ promotes multiple DNA repair pathways and therefore may perform many distinct functions. For example, several biochemical studies have documented the ability of the human Polθ polymerase domain (hereinafter referred to as Polθ-polymerase) to perform translesion synthesis in vitro, and subsequent research confirmed this activity in cells by showing that *POLQ* expression promotes replication opposite thymine glycol lesions in vivo [11,12,13]. Mammalian Polθ has also been implicated in base excision repair and somatic hypermutation during antibody maturation [14,15,16,17,18]. A recent report also indicates a role for Polθ in replication timing [19]. Although Polθ is implicated in multiple aspects of DNA metabolism, new studies performed in mice and human cells have now documented an essential role for mammalian Polθ in alt-EJ/MMEJ (microhomology-mediated end-joining) [20,21,22,23]. Here, we focus on this relatively newly discovered conserved function of Polθ which appears to be important for replication repair and the survival of cells deficient in HR.

## 2. Composition of the *POLQ* Gene 

The overall organization of Polθ encoding genes are relatively conserved among metazoans and include an A-family polymerase domain at the C-terminus and a superfamily 2 (SF2) Hel308-type helicase domain at the N-terminus. A schematic of human *POLQ* is illustrated in Figure 1. Although this type of helicase-polymerase gene fusion is unique to higher eukaryotes, multifunctional helicase-polymerase proteins have previously been identified in bacteria, archaea and viruses, and typically function in replication initiation as well as DNA repair and DNA damage tolerance [24]. Polθ encoding genes also contain a large central portion linking the helicase and polymerase domains (Figure 1). The central domain exhibits the lowest amount of sequence homology and has yet to be ascribed a specific activity or regulatory function. On the other hand, the polymerase and helicase domains exhibit relatively high sequence homology, indicating that they share common functions among different organisms. 

The C-terminal domain encodes for a unique A-family polymerase. Typical A-family polymerase members include bacterial DNA polymerase I (i.e., *E. coli* Klenow fragment), Thermus aquaticus (Taq) polymerase, Polγ, bacteriophage T7 RNA and DNA polymerases, and HIV reverse transcriptase. Although the overall sequence and structure of Polθ-polymerase is similar to Pol I type enzymes (i.e., Klenow fragment, *Bacillus* Pol I, T7 Pol I), it contains three insertion motifs whose locations within the protein are highly conserved (Figure 1) [11,12]. Insertion loop 2 in mammalian Polθ-polymerase is essential for the polymerase’s terminal transferase and translesion synthesis activities and enables it to replicate across DSBs via MMEJ (Figure 1) [11,23]. Since all other known A-family polymerases lack terminal transferase and translesion synthesis activities, loop 2 clearly confers unique characteristics onto Polθ-polymerase. Interestingly, Polθ-polymerase also contains an inactive exonuclease-like subdomain which is homologous to Klenow fragment (Figure 1) [25]. The exonuclease-like subdomain, however, is inactive due to the lack of integral residues needed for metal binding and DNA degradation. This characteristic is not unusual as other polymerases containing inactive exonuclease-like domains, such as Polα and Taq polymerase, have previously been identified [26,27].

The N-terminal helicase domain, hereinafter referred to as Polθ-helicase, is also highly conserved among vertebrates and is a member of the SF2 family of helicases (Figure 1). Polθ-helicase is most closely related to the Hel308 family of helicases which are involved in replication repair [28,29,30]. Thus, Polθ-helicase shares significant sequence homology with mammalian Hel308, also called HELQ, which is involved in replication repair and the repair of ICLs via interaction with RAD51 paralog proteins [31,32,33]. The helicase domain is also related to RecQ-type helicases which are generally involved in HR and replication repair (Figure 1) [28,34]. Although, Polθ-helicase has been shown to exhibit ATPase activity, which is maximally stimulated by single-strand DNA (ssDNA), insofar the ability of this protein to unwind double-strand DNA like other helicases has not been demonstrated [2,28]. Furthermore, whether Polθ-helicase majorly contributes to alt-EJ and collaborates with the Polθ polymerase domain during this pathway remains to be determined. Below, we review our current knowledge of Polθ-mediated alt-EJ, the respective activities of the Polθ polymerase and helicase domains, and put into perspective how these activities might contribute to alt-EJ. Finally, we discuss the potentially conserved role for Polθ-mediated alt-EJ in replication repair.

## 3. Overview of Alternative End-Joining

**Figure 2:**
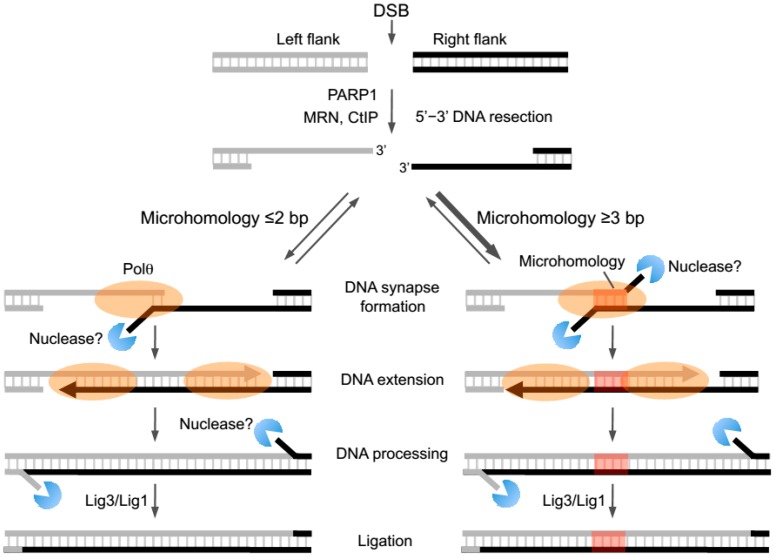
Models of Polθ-mediated end-joining. Polθ-mediated end-joining requires 5’–3’ DNA resection performed by the Mre11-Rad50-Nbs1 (MRN) nuclease complex along with CtIP which results in 3’ ssDNA overhangs. Poly (ADP ribose) polymerase I (PARP1) promotes the recruitment of Polθ to double-strand breaks (DSBs) by an undefined mechanism. Polθ-mediated end-joining may be distinguished by two different mechanisms: microhomology-mediated (**right**), and microhomology-independent (**left**). The presence of substantial microhomology (≥3 bp; red highlighted sequence) is likely to increase the half-life of the DNA synapse and therefore promote the Polθ-mediated end-joining reaction (**right**). The absence of substantial microhomology (≤2 bp) is likely to significantly reduce the efficiency of the end-joining reaction due to a shorter half-life of the DNA synapse (**left**). Following DNA synapse formation, Polθ (orange) utilizes the opposing ssDNA overhang as a template in trans to extend the DNA, which stabilizes the end-joining intermediate. Polθ may then extend the other overhang in the opposite direction, which would further stabilize the end-joining intermediate. Nucleases (blue) are likely to be required for further processing of the DNA by trimming unannealed ssDNA ends. Last, Lig1 or Lig3 is required to seal the processed DNA end-joining intermediate.

Early evidence for the alt-EJ pathway was uncovered by studies in yeast and mammalian cells almost two decades ago [35,36,37]. For example, the Jasin and Jackson laboratories identified an error-prone form of DSB repair that was independent of the Ku proteins that are essential for the major non-homologous end-joining (NHEJ) pathway [38]. The Roth lab further found that this alternative form of DSB repair acted independently of the NHEJ factor X-ray repair cross-complementing protein 4 (XRCC4) which is important for the activity of the essential NHEJ protein Ligase 4 (Lig4) [36]. Later studies found that Ligase 3 (Lig3), and Mre11-Rad50-Nbs1 (MRN) along with CtIP promote alt-EJ, indicating that resection of DNA ends specifically along the 5’ strand is needed to facilitate end-joining between opposing 3’ ssDNA overhangs (Figure 2) [39,40,41,42,43,44,45]. Support for Ligase I (Lig1) in alt-EJ also exists, demonstrating redundant roles for ligase activity in this pathway (Figure 2) [46,47]. Studies also show that Poly (ADP ribose) polymerase I (PARP1) facilitates alt-EJ repair of DSBs in multiple cell types (Figure 2) [41,48,49]. Recent reports indicate that this ubiquitous DNA repair factor is needed for the recruitment of Polθ which is an essential and central component of alt-EJ/MMEJ whose activities are reviewed in detail below (Figure 2) [21,50]. Whether PARP1 directly interacts with or modifies Polθ to facilitate its recruitment during end-joining remains unknown.

Several lines of evidence demonstrate that the respective characteristics of DNA repair junctions formed by the alt-EJ and NHEJ pathways are different which further indicates that these pathways utilize separate enzymatic activities. For example, recent studies in human cells present large data sets comparing alt-EJ and NHEJ repair junctions resulting from site-specific DSBs induced by different types of endonucleases [51]. This study distinguished chromosome translocation junctions generated by NHEJ in wild-type human cells versus those generated by alt-EJ in the same cells mutated in *LIG4* and *XRCC4*. Hence, these data provide a comprehensive documentation of deletion sizes and microhomology lengths observed at alt-EJ and NHEJ repair junctions specifically in human cells (Table 1) [51]. Deep sequencing of alt-EJ and NHEJ junctions generated in mouse embryonic fibroblasts show similar deletion and microhomology lengths [52]. The approximate range of nucleotide insertion lengths at the majority of alt-EJ and NHEJ junctions have also been documented in previous studies [22,53]. Overall, the junctions generated by NHEJ often include indels that are predominantly small in size (i.e., ≤30 base-pairs (bp) deletions, ≤5 bp insertions) (Table 1) [51]. Moreover, nucleotide insertions occur infrequently at NHEJ junctions [53]. On the other hand, indels generated by alt-EJ in human cells are typically larger in size. For instance, the majority of deletions are >30 bp in length and large deletions >100 bp are frequently observed (Table 1) [51]. Relatively long insertion tracts at repair junctions are also a general feature of alt-EJ. For example, the majority of insertions appear to be ≥3 bp in length, however, insertion tracts 5–30 bp in length are also commonly observed (Table 1) [6,20,21,22,54].

Another general characteristic of alt-EJ repair junctions is the presence of small tracts (i.e., ≥2 bp in human cells) of sequence microhomology which may promote base pairing of complementary portions of 3’ ssDNA overhangs (Figure 2, right; Table 1) [51]. Due to this feature alt-EJ is also referred to as MMEJ [23]. Current evidence, however, indicates that microhomology is not necessarily essential for end-joining. For example, multiple systems suggest that alt-EJ can proceed in the absence of microhomology or in the presence of a single 3’ terminal base pair of microhomology which is not likely to promote annealing given its relatively low melting temperature (Figure 2, left) [20,21,22,54]. It is conceivable that more extensive microhomology (i.e., >3 bp) may partially stabilize or at least slightly increase the half-life of a DNA synapse formed between 3’ ssDNA overhangs via base pairing (Figure 2, right). Transient annealing between microhomologous 3’ terminal ssDNA overhangs is likely to favor the kinetics of the next logical step of end-joining, DNA extension via polymerase activity (Figure 2, right) [23]. Such a model predicts that the alt-EJ machinery preferentially joins overhangs containing longer tracts of microhomology (Figure 2, right). Consistent with this model, initial biochemical studies of Polθ-dependent DNA end-joining demonstrate a strong preference for end-joining with increasing lengths of microhomology [23]. Recent cellular studies using an episomal end-joining model system support this model by showing that Polθ-mediated repair products are largely dependent on microhomology [52]. Interestingly, biochemical and cellular studies also demonstrate that MMEJ can occur when microhomology tracts are located internally along 3’ overhangs (Figure 2, right) [23,52]. This suggests a role for nucleases in trimming the 3’ terminus of unannealed overhangs prior to the DNA extension step (Figure 2, right). 

## 4. Polθ-Mediated Alternative End-Joining in Metazoans

The first evidence supporting a role for Polθ in the alt-EJ pathway was revealed by studies in *Drosophila melanogaster*. Chan et al. demonstrated that a form of alt-EJ that functions independently of HR and the essential NHEJ factor Ligase 4 (Lig4) is promoted by the *mus308* gene which encodes Polθ [6]. Importantly, these Polθ-mediated DNA repair products resulted in relatively large indels. The insertion and deletion lengths respectively decreased and increased in cells harboring a *mus308* mutation that significantly reduces overall levels of the polymerase. The observed insertions often included short sequence tracts that were similar or identical to flanking DNA regions, or appeared as a random sequence. These data therefore hinted at the ability of Polθ to potentially extend 3’ ssDNA overhangs by both template-dependent and template-independent mechanisms during alt-EJ.

Further evidence for Polθ-mediated alt-EJ in invertebrates was revealed in *C. elegans*. Here, Koole et al. demonstrated that cells deficient in the DOG-1/FANCJ helicase depend on Polθ-mediated end-joining to suppress large deletions occurring at highly stable G-quadruplex (G4) DNA secondary structures, which are known to arrest replication forks and cause genome instability in DOG-1 and FANCJ deficient worm and human cells, respectively [54]. Previous studies demonstrated that FANCJ promotes replication through G4 sites and unwinds these structures [55,56]. Remarkably, the *C. elegans* studies identified similar insertion tracts at repair junctions generated by Polθ as those found previously in *Drosophila* [54]. For example, the insertions again included sequence tracts that were identical to regions flanking the G4 site where DSBs likely occur [54]. Some nucleotide insertions also appeared to be generated by a template-independent mechanism [54]. Thus, this study also suggested the ability of Polθ to generate insertions by template- dependent and independent mechanisms. Polθ-mediated repair in this system also resulted in relatively large deletions and the use of sequence microhomology. Yet, Polθ-deficient cells exhibited significantly larger deletions, demonstrating a genome stabilizing function of the polymerase. Together, these seminal studies in invertebrates provided initial evidence in support of a universal signature of Polθ-mediated alt-EJ (Table 1).

Recent reports have documented an essential function for Polθ in mammalian alt-EJ/MMEJ and therefore have confirmed a conserved role for this multifunctional enzyme in this enigmatic DNA repair process [20,21,23]. The first study to demonstrate Polθ-mediated alt-EJ in mammalian cells was performed by the Wood lab which also initially characterized Polθ as a multifunctional enzyme possessing DNA dependent polymerase and ATPase activities in vitro [2,20]. Yousefzadeh et al. first demonstrated in mouse embryonic fibroblasts that Polθ promotes resistance to multiple genotoxic agents that cause DSBs [20]. They then found that Polθ promotes class-switch recombination in naive B lymphocytes when NHEJ is suppressed [20]. For example, insertions >1 bp at repair junctions were shown to be dependent on Polθ and this effect was more evident when NHEJ was suppressed by inhibition of DNA-PKcs. which is a signature of Polθ-mediated end-joining. Previous studies discovered alt-EJ as a backup form of class-switch and VDJ recombination in B cells, but did not identify a role for Polθ in this pathway [57,58,59]. Similar to observations in invertebrates, Yousefzadeh et al. found that mouse Polθ-mediated alt-EJ promotes relatively large indels at repair junctions which is consistent with a potentially universal repair signature for this pathway (Table 1) [20]. Intriguingly, Yousefzadeh et al. found that Polθ suppresses cmyc-IgH interchromosomal translocations, whereas later studies observed that the polymerase stimulates interchromosome translocations [21]. These fundamental differences in Polθ function are likely due to cell or locus specific effects.

Later studies performed by the Sfeir lab showed that Polθ promotes telomere fusions via alt-EJ in mammalian cells deficient in telomere protection proteins and NHEJ factors [21]. Importantly, in this genetic background telomere fusions were exclusively promoted by Polθ. For example, only knockdown of Polθ severely suppressed telomere fusions, whereas knockdown of Pols ν, η, μ, β, ι, and κ showed no effect [21]. Thus, these findings demonstrated a specific function for Polθ in promoting chromosome translocations. Mateos-Gomez et al. also demonstrated the ability of Polθ to promote translocations between chromosomes at non-telomere regions, and that the polymerase activity of Polθ is essential for alt-EJ, which is consistent with results from the Wood lab [20,21]. Remarkably, the DNA repair junctions observed by Mateos-Gomez et al. included indels similar to those identified in prior studies performed in mice, worms and flies [6,20,21,54]. Hence, these data along with previous studies further support an alt-EJ molecular signature at repair junctions that consists of relatively large indels compared to those formed during canonical NHEJ (Table 1) [51].

Consistent with an essential function for mammalian Polθ in alt-EJ, Kent et al. demonstrated that suppression of *POLQ* expression in human cells significantly reduces alt-EJ as detected by a commonly used chromosomally integrated green fluorescent protein (GFP) reporter system for this pathway [23]. This study also characterized the biochemical activity of the polymerase domain during a reconstituted MMEJ reaction in vitro [23]. Specifically, the purified human Polθ-polymerase was shown to promote end-joining of DNA substrates containing 3’ ssDNA overhangs with short tracts (≥2 bp) of sequence microhomology [23]. As mentioned above, these studies support the idea that alt-EJ is facilitated by sequence microhomology which is often detected at DNA repair junctions generated by this pathway in cells (Figure 2, right). Below, we further discuss the biochemical activities of Polθ and how they potentially contribute to alt-EJ and chromosome translocations in metazoans. 

## 5. Specific Activities of the Polθ Polymerase Domain

Mechanisms by which Polθ-polymerase functions in alt-EJ have been elucidated by recent biochemical studies using the purified human polymerase domain on model end-joining substrates in vitro [22,23]. As mentioned above, Polθ-polymerase was found to efficiently promote end-joining when overhangs contained >2 bp of microhomology, a process referred to as MMEJ (Figure 2, right) [23]. Less efficient end-joining was observed between substrates containing ≤2 bp of microhomology (Figure 2, left) [23]. Since Polθ-polymerase was shown to promote synapse formation between model end-joining substrates in the absence of deoxyribonucleotides (dNTPs), and this activity was enhanced when microhomology was present, the polymerase may play a role in bringing overhangs together during the initial steps of end-joining [23]. In support of this potential mechanism, gel filtration and native gel analyses demonstrated that Polθ-polymerase is capable of forming dimers and multimers, and crystallographic studies revealed tetrameric complexes of similar constructs of the protein [23,25]. Dimers or tetramers of the polymerase may facilitate DNA synapse formation. Since Polθ-polymerase was also shown to stimulate ssDNA annealing, it may help stabilize end-joining intermediates by facilitating base-pairing between microhomology regions (Figure 2, right) [23]. As discussed below, studies in *Drosophila* and mice suggest that the N-terminal helicase domain of Polθ contributes to microhomology annealing and therefore MMEJ [6,52].

Altogether, initial biochemical studies of MMEJ indicate that Polθ-polymerase promotes DNA synapse formation between 3’ ssDNA overhangs, then facilitates microhomology-mediated annealing and subsequent extension of the 3’ ssDNA terminus by using the opposing overhang as a template in trans (Figure 2, right). Interestingly, the presence of a 5’-terminal phosphate at the ssDNA/dsDNA junction increases the rate of the ssDNA extension step which suggests that Polθ-polymerase exhibits an affinity for the 5’-terminal phosphate like NHEJ polymerases (i.e., Polμ, Polλ) [23]. Lastly, Polθ-polymerase may also extend the second overhang and therefore perform two gap filling steps (Figure 2). In MMEJ reconstituted assays, Polθ-polymerase was shown to promote strand displacement during gap filling [23]. In this scenario, further processing of end-joining intermediates by nucleases and potentially other proteins would be required prior to the essential ligation step which can be performed by Lig3 or Lig1 (Figure 2) [23,42,46,47].

It is important to note that recent studies in yeast demonstrate combined roles for B-family Polδ and X-family Pol IV in MMEJ [60]. Polθ is not present in yeast and X-family polymerases Polβ and Polμ that are respectively involved in gap filling and NHEJ were not found to promote alt-EJ in mammalian cells [21]. Considering that Polδ is involved in gap filling during multiple DNA repair processes (i.e., mismatch repair, Okazaki fragment maturation), it is conceivable that this high-fidelity polymerase may also be involved in gap filling during alt-EJ/MMEJ in higher eukaryotes. Perhaps, Polθ may even hand-off the 3’ terminal end of the partially extended DNA at end-joining intermediates to Polδ for further gap filling. This would explain why mutations other than indels are not generally observed flanking alt-EJ repair junctions which would be expected if error-prone Polθ was the sole polymerase involved in this pathway. Mutations flanking alt-EJ repair junctions formed during class-switch recombination in B cells from mice, however, have been reported [20]. Thus, Polθ may preferentially perform the gap filling function in certain scenarios.

Another interesting finding from initial biochemical studies of MMEJ is the ability of Polθ-polymerase to promote end-joining even when microhomology is not present directly at the 3’ termini of overhangs [23]. For example, when short microhomology tracts were located slightly upstream from both of the 3’ termini, the polymerase was still able to perform end-joining of model substrates, albeit less efficiently [23]. This activity is consistent with the ability of Polθ-polymerase to promote relatively efficient mismatch extension [61]. Internal microhomology located further upstream was efficiently utilized when present on one of the overhangs (Figure 2, right) [23]. Since microhomologous regions are not likely to exclusively occur at ssDNA ends, these types of mechanisms would be expected to facilitate MMEJ in cells. Indeed, recent cellular studies using episomal end-joining substrates confirm that internally located microhomologous regions facilitate MMEJ (Figure 2, right) [52]. Initial biochemical studies also suggest that MMEJ activity is specific to Polθ. For example, Polθ-polymerase was replaced with different enzymes in MMEJ assays including B-family Polδ, Y-family Pols κ and η, and Klenow fragment [23]. However, only Polθ-polymerase was able to effectively promote MMEJ of model substrates, suggesting that unique motifs within the polymerase domain contribute to MMEJ [23]. 

**Figure 3:**
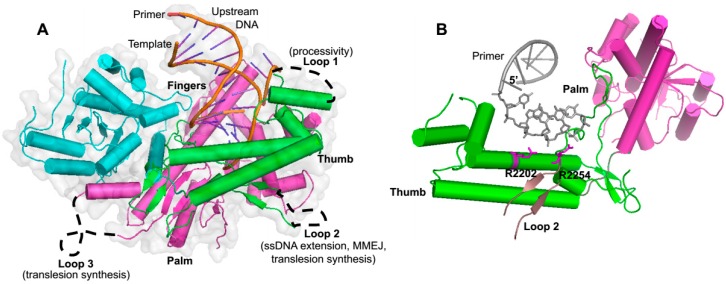
Structure of the Polθ polymerase domain. (**A**) Crystal structure of the Polθ polymerase domain in complex with a primer-template (PDB code 4X0P). Palm (purple), fingers (cyan) and thumb (green) domains are indicated. The location and reported functional roles of insertion loops, which are unstructured in the crystal, are indicated. (**B**) Close up structure highlighting the primer (grey), loop 2 (green), and conserved positively charged residues (blue: R2202, R2254) that bind the 3’ terminus of the primer (PDB code 4X0P).

As mentioned above, Polθ-polymerase shares significant sequence and structural homology to A-family polymerases such as Klenow fragment and is comprised of the three major structural elements common among polymerases referred to as thumb, palm and fingers subdomains, which overall resemble the shape of a partially closed right hand (Figure 1 and Figure 3A) [25,62]. Polθ-polymerase contains three insertion loops which are not found in other A-family polymerases, but are highly conserved among vertebrate Polθ polymerase domains (Figure 1 and Figure 3A) [25]. Insertion loop 2 is located between the palm and fingers subdomains and appears to play a direct role in activities that are likely to be important for alt-EJ. For instance, as mentioned above, loop 2 is essential for Polθ-polymerase extension of ssDNA and partial ssDNA (pssDNA), and for MMEJ activity (Figure 3A) [22,23]. This motif also contributes to the translesion synthesis activity of the polymerase (Figure 3A) [11]. X-ray crystallographic studies show that loop 2, although mostly unstructured, is positioned near the 3’ terminus of the primer in the Polθ-polymerase:primer-template complex, and a conserved positively charged residue at the N-terminus of this loop (arginine 2254) interacts with the 3’ terminal phosphate of the primer (Figure 3B) [25]. Another conserved positively charged residue (arginine/lysine) at position 2202 also binds the 3’ terminal portion of the primer (Figure 3B). Intriguingly, upon deletion of loop 2, the polymerase fails to bind pssDNA, a substrate modeled after partially resected DSBs in which Polθ-polymerase exclusively performs MMEJ on, at least in vitro [23]. Insertion loops 1 and 3 also appear as unstructured and contribute to the enzyme’s processivity and translesion synthesis activity, respectively (Figure 3A).

Biochemical studies also identified optimal substrates onto which Polθ-polymerase performs MMEJ in a minimal reconstituted reaction. For example, the polymerase was shown to require pssDNA substrates with <18 nt of ssDNA which forms the 3’ overhang portion [23]. These data suggest that the polymerase acts exclusively on DNA after it is initially resected by the MRN complex presumably along with CtIP, which is likely to generate relatively short 3’ overhangs (i.e., 15–20 nt) like its yeast counterpart, the Mre11-Rad50-Xrs2-Sae2 complex (Figure 2) [63]. Recent cellular studies, however, indicate that Polθ is able to function on significantly longer overhangs which suggests the possibility that it is specifically recruited to ssDNA by another factor, or that the full-length protein behaves differently on ssDNA [52].

Although biochemical studies have provided important insight into how Polθ-polymerase promotes MMEJ, cellular studies performed in mice, flies and worms have clearly demonstrated the ability of Polθ to generate nucleotide insertions at alt-EJ repair junctions [6,20,21,22,52,54]. In many cases small insertion tracts appear to be copied from regions flanking the breaks resulting in small (~3–10 bp) sequence duplications. In other cases, insertions show no relation to nearby regions and therefore appear as random sequence. Cellular studies have also shown that although repair junctions formed by alt-EJ can be associated with microhomology, this process does not necessarily rely on homologous sequences between overhangs [22]. For example, as mentioned above frequently only 1 bp of microhomology is observed at junctions which is not sufficient for facilitating annealing due to the low melting temperate of a single base pair [20,22,54]. These cellular studies therefore have demonstrated that Polθ can also promote end-joining when little or no microhomology is present along ssDNA overhangs (Figure 2, left).

**Figure 4:**
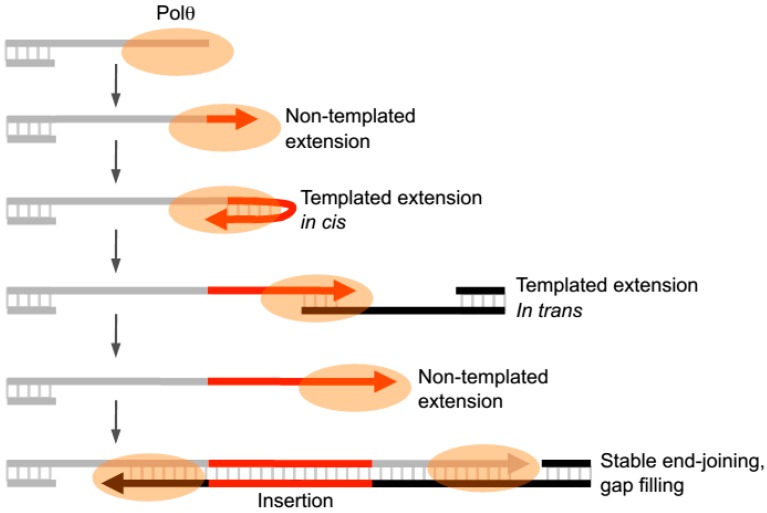
Model of Polθ-polymerase terminal transferase activity during end-joining. Schematic representation of Polθ-polymerase switching between three different terminal transferase activities during end-joining. Polθ-polymerase performs non-templated ssDNA extension, templated extension in cis, and templated extension in trans, and spontaneously switches between these activities during end-joining which results in a combination of templated and non-templated nucleotide insertions at repair junctions.

Recent in vitro studies have now revealed that Polθ-polymerase can also promote end-joining in the absence of significant microhomology (i.e., <2 bp) and generate insertions during this process by utilizing templated and non-templated mechanisms of terminal transferase activity (Figure 4) [22]. The presence of manganese (Mn^2+^), even at low levels (i.e., 50–200 µM), was shown to stimulate the enzyme’s template-independent terminal transferase activity and therefore allows the polymerase to transfer nucleotides to the 3’ terminus of ssDNA in a random manner [22]. In the presence of only magnesium (Mg^2+^), the polymerase was shown to primarily extend ssDNA by using the initially bound ssDNA as a template in cis or by utilizing another ssDNA substrate as a template in trans [22]. These extension products therefore contain sequence tracts that are mostly complementary to the ssDNA sequence present in the reaction. These studies also showed that the ratio of Mn^2+^ to Mg^2+^ modulates the balance between template-dependent and independent terminal transferase mechanisms [22]. For example, a high ratio of Mg^2+^ to Mn^2+^ stimulates template-dependent activity, whereas low ratios of Mg^2+^ to Mn^2+^ promotes template-independent activity. Since Polθ appears to generate random and templated nucleotide insertions at repair junctions in multiple organisms, Mn^2+^ likely acts as a co-factor for the polymerase in vivo. Interestingly, Mn^2+^ is a necessary co-factor for Mre11 which is involved in the DNA resection step required for both alt-EJ and HR [44,63]. Thus, even low amounts of intracellular manganese (50–200 µM) are likely to promote particular enzymatic activities in vivo. 

Intriguingly, Polθ-polymerase was shown to spontaneously switch between template- dependent and independent modes of DNA synthesis when Mg^2+^ is present at 10-fold higher concentrations than Mn^2+^ which models cellular conditions [22]. For example, the polymerase was shown to oscillate between the following three modes of terminal transferase activity: non-templated extension, templated extension in cis (also called snap-back replication), and templated extension in trans (Figure 4) [22]. Switching between these distinct terminal transferase activities was evident at DNA repair junctions formed during partially reconstituted alt-EJ in vitro in the presence of Polθ-polymerase and Lig3, as well as at alt-EJ junctions formed in vivo [22]. For instance, short tracks of sequence inserts appeared to be generated by the three different terminal transferase mechanisms. Moreover, the polymerase was shown to act with high processivity during ssDNA extension and end-joining in vitro [22]. Thus the enzyme apprears to switch between three different terminal transferase activities without dissociating from the initial DNA substrate during end-joining (Figure 4).

Although the structural basis for how Polθ-polymerase facilitates end-joining remains to be determined, loop 2 appears to be important for this activity (Figure 3A). As mentioned above, loop 2 is essential for the enzyme’s ability to extend ssDNA. Although loop 2 is highly conserved in vertebrates, it is significantly shorter and lacks sequence similarity in invertebrates (i.e., *C. elegans*, *Drosophila*). Nevertheless, both invertebrate and vertebrate Polθ promote similar insertion mutations during alt-EJ [6,20,21,22,54]. Thus, the conserved location of this loop may be more important than the actual structure which is unresolved in recently published human Polθ-polymerase crystals and therefore appears to be flexible in nature (Figure 3A) [25]. Since deletion of loop 2 causes the polymerase domain to be unstable, it may promote proper folding of the enzyme [11]. Future X-ray crystallographic and biochemical studies are needed to further understand how the unique functional and structural characteristics of Polθ-polymerase contribute to end-joining.

## 6. Specific Activities of the Polθ Helicase Domain

While the biochemical activities of the polymerase domain are emerging, the respective functions of the helicase and central domains are still largely unknown. As mentioned above, the presence of both a helicase and polymerase domain, separated by a large central domain as found in Polθ, is unique among DNA polymerases in eukaryotes (Figure 1). Polθ-helicase is a member of the SF2 helicase family which exhibits DNA dependent ATPase activity and utilizes the energy from ATP hydrolysis to translocate along either double- or single- strand DNA depending on the sub-family type [64,65,66,67]. For example, Polθ-helicase shares significant (30%) sequence homology with Hel308 (Figure 1) [28]. Although translocase activity by Polθ-helicase has not been demonstrated, Hel308 translocates along ssDNA with 3’–5’ polarity in an ATP-dependent manner [29,33,68,69]. This activity, along with a β-hairpin loop, enables unwinding of dsDNA adjacent to a 3’ ssDNA overhang and may therefore be important for unwinding lagging strands at replication forks during replication repair [29,33]. Polθ-helicase is also similar in sequence to RecQ-subtype SF2 family helicases. For example, human Polθ-helicase shares 18% sequence homology with RECQL5, RECQ4 and Bloom’s helicases (Figure 1). These and other RecQ-type helicases, such as those in bacteria, are involved in many aspects of replication repair and HR, pathways in which Polθ-helicase is implicated [34,70,71,72].

**Figure 5:**
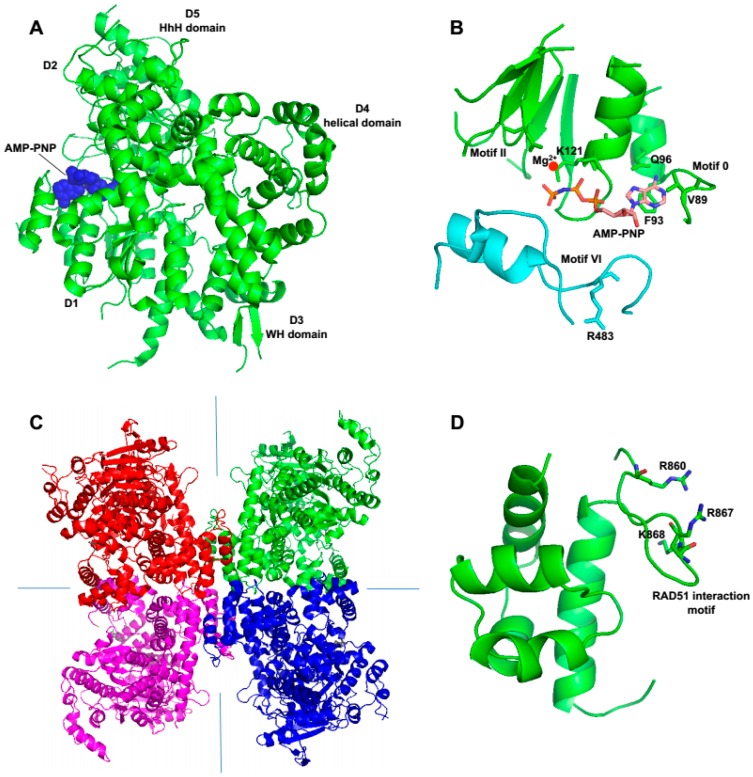
Structure of the Polθ helicase domain. (**A**) Crystal structure of Polθ-helicase (PDB code 5AGA). The location of helicase domains D1–D5 and AMP-PNP are indicated. (**B**) Close up structure of the conserved nucleotide binding domain in complex with AMP-PNP (PDB code 5AGA). (**C**) Structure of the tetrameric form of Polθ-helicase. Individual Polθ-helicase monomers are represented in blue, green, red and cyan (PDB code 5A9J). Symmetry axis is indicated as blue lines. (**D**) Close up structure of the RAD51 binding site (residues 861–868) (PDB code 5A9J).

Recent X-ray crystallographic and biochemical studies show that human Polθ-helicase possesses characteristics that are common among many SF2 helicases, such as archaea Hel308 and those in the RecQ-subclass [28,50]. For example, similar to RecQ-type helicases and archaea Hel308, Polθ-helicase binds relatively tightly to ssDNA, partial ssDNA, and replication fork substrates [28]. However, it binds less tightly to dsDNA, suggesting it preferentially functions on ssDNA [28]. Polθ-helicase structures show that it includes a highly conserved nucleotide-binding site and core helicase motifs, similar to those found in Hel308 and RecQ-type enzymes (Figure 1 and Figure 5A,B) [28,34]. This structural similarity is expected based on the high sequence conservation between the respective motifs in these enzymes (Figure 1). Biochemical assays also demonstrate that the ATPase activity of Polθ-helicase is more highly stimulated by ssDNA compared to dsDNA [50]. RecQ and Hel308 type helicases also appear to exhibit enhanced ATPase activity on ssDNA, which demonstrates a shared ssDNA preference for these enzymes [29,68,73]. RecQ helicases such as human RECQL5 and Bloom’s syndrome helicase (BLM) have also been shown to bind RAD51 recombinase which contributes to their anti-recombinase activity that counteracts RAD51-mediated D-loops [72,74]. Polθ-helicase also interacts with RAD51 and exhibits anti-recombinase activity which is further discussed below.

Considering that Polθ-helicase exhibits high sequence similarity to archaea Hel308, Newman et al. examined the structural conservation between these proteins by superimposing their respective structural motifs [28]. Several of the structural domains involved in helicase activity, as well as ssDNA binding motifs, nucleotide-binding, and helicase motifs I–IV superimpose well, likely indicating similar functions [28]. However, it is important to note that the overall Polθ-helicase structure did not align well with Hel308 [28]. To overcome this, the individual motifs of Polθ-helicase were superimposed separately. This overall difference in structure suggests the possibility that these helicases exhibit different functions. For example, unlike Hel308 and RecQ-type helicases, insofar Polθ-helicase has not been shown to exhibit DNA unwinding activity [2,28]. 

The reasons for the lack of observed DNA unwinding by Polθ-helicase have yet to be determined. One such possible explanation is that this activity may be dependent on additional cofactors or specific substrates. However, most known functioning helicases require only ATP as a cofactor and are able to function on several types of substrates. From a structural perspective, Polθ-helicase is not void of any conserved SF2-type helicase motifs which could have explained the apparent lack of helicase activity. Since Polθ-helicase possesses an active ATPase core domain, this can also be excluded as a potential cause for the absence of helicase activity. Considering that related SF2 helicases such as RECQL5 exhibit ssDNA annealing activity, Polθ-helicase may similarly perform this function which could conceivably mask its ability to unwind DNA in biochemical reactions [75]. Indeed, as discussed below studies performed in *Drosophila* and mice support an annealing role for Polθ-helicase in MMEJ [6,52]. 

Perhaps the most unique structural feature of Polθ-helicase is its ability to form a tetrameric arrangement which is observed in crystal structures as well as in solution (Figure 5C) [28]. A large hydrophobic surface along with interfaces that form polar contacts appear to enable this specific tetrameric formation [28]. Although this multimeric structural organization is not conserved across the Hel308 family it has been observed for RECQ1 helicase [28,70]. Interestingly, the polymerase domain of Polθ also forms multimeric complexes in crystals and solution [23,25]. Thus, the possibility exists that multimeric complexes of Polθ facilitate DNA synapse formation which is required for end-joining and chromosome translocations. 

**Figure 6:**
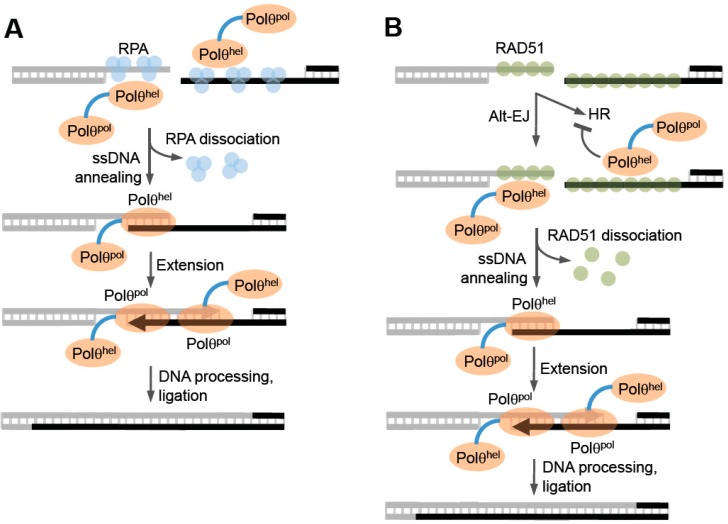
Models of Polθ-helicase activity during end-joining. (**A**) Model of end-joining involving Polθ-helicase ssDNA annealing activity. Polθ-helicase may interact with RPA and promote ssDNA annealing at microhomologous regions which is likely to facilitate end-joining. After annealing and DNA synapse formation, the polymerase domain performs ssDNA extension by utilizing the opposing overhang as a template in trans. (**B**) Model of end-joining involving Polθ-helicase anti-recombinase activity. Alt-EJ and HR share the same resection mechanism and therefore compete during S and G2 cell-cycle phases. Polθ-helicase may dissociate RAD51 filaments, then perform annealing which would enable Polθ-polymerase extension and subsequent steps of end-joining.

Genetic studies performed in *Drosophila* provided initial insight into the potential function of Polθ-helicase in alt-EJ. Chan et al. demonstrated that cells harboring mutations within the conserved helicase domain that were suggested to inactivate its ATPase activity produce Polθ-mediated DNA repair junctions with less microhomology compared to wild-type cells [6]. This suggests that Polθ-helicase may promote annealing of overhangs at microhomologous regions (Figure 6, left). Consistent with this, recent cellular studies in mice, employing an episomal model end-joining substrate, support a possible ssDNA annealing function for the helicase domain [52]. Instead, it may function as an annealing helicase like human RECQL5 or HepA-related protein (HARP) which is a SWI2/SNF2 type ATP molecular motor protein within the SF2 superfamily [76,77]. HARP was shown to promote ATP-dependent ssDNA annealing in the presence of RPA, which blocks spontaneous annealing of homologous ssDNA [76]. Considering that RPA has been shown to suppress MMEJ in yeast, annealing activity by Polθ-helicase in the presence of RPA would likely stimulate alt-EJ of ssDNA overhangs containing microhomology (Figure 6, left) [78]. Such an activity is akin to RAD52-mediated ssDNA annealing which overcomes RPA suppression of complementary DNA duplex formation, albeit in an ATP independent manner. Future studies are needed to determine whether Polθ-helicase functions like HARP and promotes annealing during alt-EJ, and whether this putative activity might involve active or passive dissociation of RPA from ssDNA.

HARP and other SF2 helicases such as those within the Hel308 and RecQ subclasses are generally characterized as replication repair factors due to their involvement in the repair of collapsed or stalled replication forks [29,69,77]. Since recent studies show that Polθ majorly influences replication fork speed and timing, its helicase domain may also play a direct role in replication fork repair [19,50]. In support of this idea, multiple lines of evidence support a role for alt-EJ in replication repair (discussed below).

Multiple pathways function to repair collapsed replication forks and DSBs during S and G2 phases including alt-EJ [44,79,80,81]. Thus, it is important to consider the potential competition between Polθ-mediated repair and other pathways. Polθ-helicase has been shown to suppress homologous recombination (HR) which is the major DNA break repair pathway during S and G2 phases (Figure 6, right) [50]. HR is considered highly accurate due to its ability to use homologous DNA, for example the sister chromatid, as a template for repair [82]. This pathway requires the strand invasion function of RAD51 whose activity and loading on ssDNA is highly regulated by many factors including BRCA1, BRCA2, and several RAD51 mediator proteins [79,81,82,83]. To ensure high-fidelity DNA break repair during replication, HR factors and complex regulatory mechanisms have evolved to suppress less accurate DNA repair pathways such as NHEJ and SSA in S and G2 phases [84,85]. How cells regulate the choice between accurate HR and alt-EJ which is highly error-prone is an active area of research that will likely lead to a better understanding of how cells maintain genome integrity and suppress tumorigenesis.

The generation of an ssDNA overhang was originally thought to represent commitment to HR directed repair. However, evidence indicates that this initial end resection is shared between HR and alt-EJ [44]. For example, both alt-EJ and HR share the requirements for MRN and CtIP which are necessary for the initial end resection step involved in the repair of DSBs [44]. When both alt-EJ and HR are available and therefore potentially in competition, alt-EJ was reported to occur at a rate of 10%–20% of HR [44]. Thus, although it was originally proposed that alt-EJ served primarily as a back-up mechanism when other repair pathways such as HR and NHEJ were absent or deficient, it is now accepted that alt-EJ can occur when NHEJ and HR are fully functional [44]. In fact, new studies indicate that alt-EJ plays a major role in DSB repair during vertebrate development [86].

Since Polθ-helicase has been shown to negatively regulate HR, it appears to directly influence the choice between HR and alt-EJ (Figure 6, right) [50]. Ceccaldi et al. directly examined the relationship between Polθ expression and HR and found that suppression of Polθ significantly increased HR levels [50]. Similar increases in HR were previously observed following suppression of anti-recombination helicases PARI and BLM [50,74,87]. Consistent with a potential role as an anti-recombination factor, portions of the helicase and central domain of Polθ were shown to directly interact with RAD51 (Figure 1 and Figure 5D) [50]. Further data demonstrated that Polθ-helicase (residues 1–1,000) suppresses HR and RAD51 foci formation following ionizing radiation [50]. Finally, direct evidence in support of anti-recombination activity was revealed by the ability of the helicase to counteract RAD51-ssDNA filaments (Figure 6, right) [50]. In contrast, mutant versions of Polθ-helicase defective in either ATPase activity or RAD51 binding failed to antagonize RAD51 activity [50]. Data was also presented that suggests that Polθ-deficient cells fail to keep RAD51 in check and therefore become toxic due to hyper-recombination [50]. Consistent with this scenario, inactivation of the yeast SF1 helicase Srs2, which exhibits anti-recombinase activity, results in hyper-recombination that is toxic to the cell [88,89,90]. Since Polθ-helicase utilizes its anti-recombinase activity to suppress HR which competes with alt-EJ, the possibility exists that the helicase domain dissociates RAD51 from ssDNA for the purpose of promoting end-joining (Figure 6, right). Notably, the observed suppression of HR by Polθ may be specific to vertebrates since currently no evidence for this phenomenon exists in invertebrate systems. Furthermore, recent studies in mice failed to observe an effect of Polθ expression in a gene targeting HR assay [52]. Thus, further studies may be required to confirm a regulatory function for Polθ in HR.

## 7. Evidence for Polθ-Mediated Alt-EJ in Replication Repair

**Figure 7:**
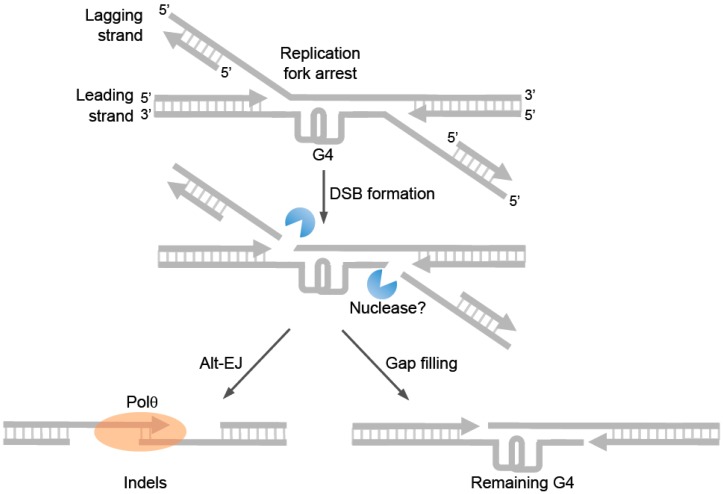
Model of Polθ-mediated end-joining of arrested replication forks. Model of Polθ-mediated end-joining of replication forks arrested at a G-quadruplex (G4). Following the arrest of divergent replication forks at G4, nucleases may cleave the lagging strands which would generate DSBs with 3’ ssDNA overhangs. Polθ may then promote DSB repair via alt-EJ (**left**). Gap filling on the leading strands may leave the G4 intact (**right**).

It has been well documented that Polθ repairs DSBs via the alt-EJ pathway, and mounting evidence suggests that this process is exclusively activated during S-phase and therefore likely involved in replication repair. Studies in *C. elegans* probably provide the most convincing evidence in support of alt-EJ in the repair of replication forks. For example, as mentioned above cells deficient in DOG-1 helicase (FANCJ ortholog) rely on Polθ-mediated repair to prevent large deletions from occurring at G4 sites (Figure 7) [54]. Since DOG-1/FANCJ normally promotes replication past G4 sites and suppresses genome instability, error-prone repair of collapsed replication forks at G4 sites probably accounts for the observed genome instability in DOG-1 deficient cells. Consistent with this idea, Koole et al. demonstrated that relatively large indels characteristic of alt-EJ were generated at G4 sites in a Polθ dependent manner in DOG-1 deficient cells (Figure 7) [54]. Hence, these seminal studies reveal a major mechanism of replication repair via Polθ-mediated alt-EJ in invertebrates.

Truong et al. demonstrated that MMEJ is activated by cyclin-dependent kinase activity in mammalian cells, and that this pathway is significantly upregulated in S-phase cells [44]. These authors also presented data suggesting that MMEJ might preferentially repair breaks associated with arrested replication forks [44]. For example, significant stimulation of MMEJ but not HR repair of chromosomally integrated GFP reporters was observed following hydroxyurea treatment or suppression of factors that are important for stabilizing replication forks such as ATR and Timeless [44]. As mentioned above, this study also demonstrated that MMEJ and HR share the same initial DSB resection mechanism promoted by MRN and CtIP [44]. Consistent with this, HR was shown to suppress MMEJ in recent studies [91]. Considering that resection of DSBs during S and G2 cell cycle phases is licensed by cyclin-dependent kinase phosphorylation of CtIP which is essential for alt-EJ/MMEJ, these data further support the idea that alt-EJ/MMEJ exclusively functions during replication [85]. Consistent with this idea, mechanistic studies performed in mouse embryonic fibroblasts show that Polθ promotes end-joining of episomal substrates resembling collapsed replication forks [52]. 

Further evidence supporting a significant role for alt-EJ in DSB repair during S-phase, and thus replication repair, has been revealed by recent studies in HR deficient cells. For example, Ceccaldi et al. demonstrated a synthetic lethal relationship between HR and Polθ which indicates a compensatory role for alt-EJ in repairing DSBs during S and G2 cell cycle phases, such as those caused by replicative stress [44,50]. Loss of both HR and Polθ-mediated repair in *Fancd2*/*Polq* double knockout mice resulted in nearly full lethality [50]. In contrast, mice with knockouts of either *Fancd2* or *Polq* alone were viable [50]. Consistent with these findings, a separate study demonstrated that Polθ expression is essential for the proliferation of breast cancer cells deficient in BRCA1 [21]. Ceccaldi et al. also showed that HR-deficient cells, including those that are defective in BRCA1 or BRCA2, are hypersensitive to PARP1 inhibitors when Polθ expression is suppressed [50]. PARP1 inhibitors are thought to promote replication dependent DNA breaks which are primarily repaired by HR and therefore cause synthetically lethality when administered to HR deficient cells [92,93,94]. Altogether, these results demonstrate the importance of the proper activity of at least one of these DNA repair mechanisms, for example HR or Polθ-mediated repair, for cellular survival, especially in the face of DNA damage that arrests replication forks. Remarkably, recent studies also demonstrate that Polθ-mediated repair is important for the proliferation of mouse cells defective in NHEJ [52]. Hence, cells deficient in either of the major canonical DSB repair pathways, HR or NHEJ, are hyper-dependent on Polθ.

Further studies performed in mammalian cells provide additional support for Polθ in replication repair. For example, Ceccaldi et al. demonstrated that replication fork velocity is decreased in cells deficient in Polθ, even in the absence of exogenous DNA damaging agents [50]. Replication progression after hydroxyurea treatment which causes replication fork arrest was also severely impaired [50]. Hence, these data directly support a role for Polθ in promoting the progression or repair of replication forks. However, considering that this multifunctional protein also promotes translesion synthesis and is involved in replication timing, it remains to be determined whether and how alt-EJ specifically contributes to replication repair in mammalian cells. Nevertheless, because cells deficient in HR and Polθ are synthetic lethal, and HR and alt-EJ utilize the same resection initiation mechanism during S-phase, current evidence already provides strong support for an important role for Polθ-mediated alt-EJ in replication repair in mammalian cells.

## 8. Conclusions

In summary, Polθ is a multifunctional polymerase-helicase protein that promotes alt-EJ/MMEJ repair of DSBs, while generating a distinct DNA repair signature characterized by the presence of large indels and microhomology flanking the repair junction. Although, alt-EJ was first believed to exclusively act as a backup DNA repair pathway when NHEJ was unavailable, recent research demonstrates that alt-EJ is activated during S and G2 cell-cycle phases and functions, albeit at low levels, in the presence of major canonical NHEJ and HR pathways. Yet when HR or NHEJ are deficient, cells become hyper-dependent on Polθ-mediated repair for their proliferation. Future studies aimed at identifying the specific activities of the polymerase and helicase domains will aid in the development of drug inhibitors of this multi-functional enzyme that promotes the survival of HR-deficient cancer cells. 

## Figures and Tables

**Table 1:** Characteristics of end-joining repair signatures. The majority of DNA deletion and microhomology lengths observed at non-homologous end-joining (NHEJ) and alt-EJ repair junctions were reported in reference 51. The majority of insertion lengths observed at NHEJ and alt-EJ repair junctions were reported in references 52 and 22, respectively. The relative frequency of events observed at junctions is indicated by the following symbols: (+) low frequency; (+++) high frequency; (++) moderate frequency.

	Deletions (bp)	Insertions (bp)	Microhomology (bp)
Alt-EJ	>30 (+++)	≤30 (+++)	≥2 (++)
NHEJ	≤30 (++)	≤5 (+)	≤2 (+)

## References

[1] Shima N., Hartford S.A., Duffy T., Wilson L.A., Schimenti K.J., Schimenti J.C. (2003). Phenotype-based identification of mouse chromosome instability mutants. Genetics.

[2] Seki M., Marini F., Wood R.D. (2003). POLQ (Pol theta), a DNA polymerase and DNA-dependent ATPase in human cells. Nucleic Acids Res..

[3] Shima N., Munroe R.J., Schimenti J.C. (2004). The mouse genomic instability mutation chaos1 is an allele of Polq that exhibits genetic interaction with Atm. Mol. Cell. Biol..

[4] Goff J.P., Shields D.S., Seki M., Choi S., Epperly M.W., Dixon T., Wang H., Bakkenist C.J., Dertinger S.D., Torous D.K. (2009). Lack of DNA polymerase theta (POLQ) radiosensitizes bone marrow stromal cells in vitro and increases reticulocyte micronuclei after total-body irradiation. Radiat. Res..

[5] Higgins G.S., Prevo R., Lee Y.F., Helleday T., Muschel R.J., Taylor S., Yoshimura M., Hickson I.D., Bernhard E.J., McKenna W.G. (2010). A small interfering RNA screen of genes involved in DNA repair identifies tumor-specific radiosensitization by POLQ knockdown. Cancer Res..

[6] Chan S.H., Yu A.M., McVey M. (2010). Dual roles for DNA polymerase theta in alternative end-joining repair of double-strand breaks in Drosophila. PLoS Genet..

[7] Boyd J.B., Sakaguchi K., Harris P.V. (1990). mus308 mutants of Drosophila exhibit hypersensitivity to DNA cross-linking agents and are defective in a deoxyribonuclease. Genetics.

[8] Muzzini D.M., Plevani P., Boulton S.J., Cassata G., Marini F. (2008). Caenorhabditis elegans POLQ-1 and HEL-308 function in two distinct DNA interstrand cross-link repair pathways. DNA Repair.

[9] Harris P.V., Mazina O.M., Leonhardt E.A., Case R.B., Boyd J.B., Burtis K.C. (1996). Molecular cloning of Drosophila mus308, a gene involved in DNA cross-link repair with homology to prokaryotic DNA polymerase I genes. Mol. Cell. Biol..

[10] Leonhardt E.A., Henderson D.S., Rinehart J.E., Boyd J.B. (1993). Characterization of the mus308 gene in Drosophila melanogaster. Genetics.

[11] Hogg M., Seki M., Wood R.D., Doublie S., Wallace S.S. (2011). Lesion bypass activity of DNA polymerase theta (POLQ) is an intrinsic property of the pol domain and depends on unique sequence inserts. J. Mol. Biol..

[12] Seki M., Masutani C., Yang L.W., Schuffert A., Iwai S., Bahar I., Wood R.D. (2004). High-efficiency bypass of DNA damage by human DNA polymerase Q. EMBO J..

[13] Yoon J.H., Roy Choudhury J., Park J., Prakash S., Prakash L. (2014). A Role for DNA Polymerase theta in Promoting Replication through Oxidative DNA Lesion, Thymine Glycol, in Human Cells. J. Biol. Chem..

[14] Yoshimura M., Kohzaki M., Nakamura J., Asagoshi K., Sonoda E., Hou E., Prasad R., Wilson S.H., Tano K., Yasui A. (2006). Vertebrate POLQ and POLbeta cooperate in base excision repair of oxidative DNA damage. Mol. Cell.

[15] Masuda K., Ouchida R., Takeuchi A., Saito T., Koseki H., Kawamura K., Tagawa M., Tokuhisa T., Azuma T., Jiyang O. (2005). DNA polymerase theta contributes to the generation of C/G mutations during somatic hypermutation of Ig genes. Proc. Natl. Acad. Sci. USA.

[16] Kohzaki M., Nishihara K., Hirota K., Sonoda E., Yoshimura M., Ekino S., Butler J.E., Watanabe M., Halazonetis T.D., Takeda S. (2010). DNA polymerases nu and theta are required for efficient immunoglobulin V gene diversification in chicken. J. Cell Biol..

[17] Masuda K., Ouchida R., Hikida M., Kurosaki T., Yokoi M., Masutani C., Seki M., Wood R.D., Hanaoka F., Jiyang O. (2007). DNA polymerases eta and theta function in the same genetic pathway to generate mutations at A/T during somatic hypermutation of Ig genes. J. Biol. Chem..

[18] Prasad R., Longley M.J., Sharief F.S., Hou E.W., Copeland W.C., Wilson S.H. (2009). Human DNA polymerase theta possesses 5'-dRP lyase activity and functions in single-nucleotide base excision repair in vitro. Nucleic Acids Res..

[19] Fernandez-Vidal A., Guitton-Sert L., Cadoret J.C., Drac M., Schwob E., Baldacci G., Cazaux C., Hoffmann J.S. (2014). A role for DNA polymerase theta in the timing of DNA replication. Nat. Commun..

[20] Yousefzadeh M.J., Wyatt D.W., Takata K.I., Mu Y., Hensley S.C., Tomida J., Bylund G.O., Doublié S., Johansson E., Ramsden D.A. (2014). Mechanism of suppression of chromosomal instability by DNA polymerase POLQ. PLoS Genet..

[21] Mateos-Gomez P.A., Gong F., Nair N., Miller K.M., Lazzerini-Denchi E., Sfeir A. (2015). Mammalian polymerase theta promotes alternative NHEJ and suppresses recombination. Nature.

[22] Kent T., Mateos-Gomez P.A., Sfeir A., Pomerantz R.T. (2016). Polymerase theta is a robust terminal transferase that oscillates between three different mechanisms during end-joining. Elife.

[23] Kent T., Chandramouly G., McDevitt S.M., Ozdemir A.Y., Pomerantz R.T. (2015). Mechanism of microhomology-mediated end-joining promoted by human DNA polymerase θ. Nat. Struct. Mol. Biol..

[24] Guilliam T.A., Keen B.A., Brissett N.C., Doherty A.J. (2015). Primase-polymerases are a functionally diverse superfamily of replication and repair enzymes. Nucleic Acids Res..

[25] Zahn K.E., Averill A.M., Aller P., Wood R.D., Doublie S. (2015). Human DNA polymerase theta grasps the primer terminus to mediate DNA repair. Nat. Struct. Mol. Biol..

[26] Coloma J., Johnson R.E., Prakash L., Prakash S., Aggarwal A.K. (2016). Human DNA polymerase alpha in binary complex with a DNA:DNA template-primer. Sci. Rep..

[27] Kim Y., Eom S.H., Wang J., Lee D.S., Suh S.W., Steitz T.A. (1995). Crystal structure of Thermus aquaticus DNA polymerase. Nature.

[28] Newman J.A., Cooper C.D., Aitkenhead H., Gileadi O. (2015). Structure of the Helicase Domain of DNA Polymerase Theta Reveals a Possible Role in the Microhomology-Mediated End-Joining Pathway. Structure.

[29] Tafel A.A., Wu L., McHugh P.J. (2011). Human HEL308 localizes to damaged replication forks and unwinds lagging strand structures. J. Biol. Chem..

[30] Woodman I.L., Brammer K., Bolt E.L. (2011). Physical interaction between archaeal DNA repair helicase Hel308 and Replication Protein A (RPA). DNA Repair.

[31] Takata K., Reh S., Tomida J., Person M.D., Wood R.D. (2013). Human DNA helicase HELQ participates in DNA interstrand crosslink tolerance with ATR and RAD51 paralogs. Nat. Commun..

[32] Adelman C.A., Lolo R.L., Birkbak N.J., Murina O., Matsuzaki K., Horejsi Z., Parmar K., Borel V., Skehel J.M., Stamp G. (2013). HELQ promotes RAD51 paralogue-dependent repair to avert germ cell loss and tumorigenesis. Nature.

[33] Buttner K., Nehring S., Hopfner K.P. (2007). Structural basis for DNA duplex separation by a superfamily-2 helicase. Nat. Struct. Mol. Biol..

[34] Hickson I.D. (2003). RecQ helicases: Caretakers of the genome. Nat. Rev. Cancer.

[35] Liang F., Jasin M. (1996). Ku80-deficient cells exhibit excess degradation of extrachromosomal DNA. J. Biol. Chem..

[36] Kabotyanski E.B., Gomelsky L., Han J.O., Stamato T.D., Roth D.B. (1998). Double-strand break repair in Ku86- and XRCC4-deficient cells. Nucleic Acids Res..

[37] Boulton S.J., Jackson S.P. (1996). Saccharomyces cerevisiae Ku70 potentiates illegitimate DNA double-strand break repair and serves as a barrier to error-prone DNA repair pathways. EMBO J..

[38] Deriano L., Roth D.B. (2013). Modernizing the nonhomologous end-joining repertoire: Alternative and classical NHEJ share the stage. Annu. Rev. Genet..

[39] Xie A., Kwok A., Scully R. (2009). Role of mammalian Mre11 in classical and alternative nonhomologous end joining. Nat. Struct. Mol. Biol..

[40] Lee-Theilen M., Matthews A.J., Kelly D., Zheng S., Chaudhuri J. (2011). CtIP promotes microhomology-mediated alternative end joining during class-switch recombination. Nat. Struct. Mol. Biol..

[41] Audebert M., Salles B., Calsou P. (2004). Involvement of poly(ADP-ribose) polymerase-1 and XRCC1/DNA ligase III in an alternative route for DNA double-strand breaks rejoining. J. Biol. Chem..

[42] Simsek D., Brunet E., Wong S.Y.W., Katyal S., Gao Y., McKinnon P.J., Lou J., Zhang L., Li J., Rebar E.J. (2011). DNA ligase III promotes alternative nonhomologous end-joining during chromosomal translocation formation. PLoS Genet..

[43] Zhang Y., Jasin M. (2011). An essential role for CtIP in chromosomal translocation formation through an alternative end-joining pathway. Nat. Struct. Mol. Biol..

[44] Truong L.N., Li Y., Shi L.Z., Hwang P.Y.H., He J., Wang H., Razavian N., Berns M.W., Wu X. (2013). Microhomology-mediated End Joining and Homologous Recombination share the initial end resection step to repair DNA double-strand breaks in mammalian cells. Proc. Natl. Acad. Sci. USA.

[45] Deriano L., Stracker T.H., Baker A., Petrini J.H., Roth D.B. (2009). Roles for NBS1 in alternative nonhomologous end-joining of V(D)J recombination intermediates. Mol. Cell.

[46] Lu G., Duan J., Shu S., Wang X., Gao L., Guo J., Zhang Y. (2016). Ligase I and ligase III mediate the DNA double-strand break ligation in alternative end-joining. Proc. Natl. Acad. Sci. USA.

[47] Masani S., Han L., Meek K., Yu K. (2016). Redundant function of DNA ligase 1 and 3 in alternative end-joining during immunoglobulin class switch recombination. Proc. Natl. Acad. Sci. USA.

[48] Wang M., Wu W., Wu W., Rosidi B., Zhang L., Wang H., Iliakis G. (2006). PARP-1 and Ku compete for repair of DNA double strand breaks by distinct NHEJ pathways. Nucleic Acids Res..

[49] Mansour W.Y., Rhein T., Dahm-Daphi J. (2010). The alternative end-joining pathway for repair of DNA double-strand breaks requires PARP1 but is not dependent upon microhomologies. Nucleic Acids Res..

[50] Ceccaldi R., Liu J.C., Amunugama R., Hajdu I., Primack B., Petalcorin M.I., O’Connor K.W., Konstantinopoulos P.A., Elledge S.J., Boulton S.J. (2015). Homologous-recombination-deficient tumours are dependent on Polθ-mediated repair. Nature.

[51] Ghezraoui H., Piganeau M., Renouf B., Renaud J.B., Sallmyr A., Ruis B., Oh S., Tomkinson A.E., Hendrickson E.A., Giovannangeli C. (2014). Chromosomal translocations in human cells are generated by canonical nonhomologous end-joining. Mol. Cell.

[52] Wyatt D.W., Feng W., Conlin M.P., Yousefzadeh M.J., Roberts S.A., Mieczkowski P., Wood R.D., Gupta G.P., Ramsden D.A. (2016). Essential Roles for Polymerase theta-Mediated End Joining in the Repair of Chromosome Breaks. Mol. Cell.

[53] Rebuzzini P., Khoriauli L., Azzalin C.M., Magnani E., Mondello C., Giulotto E. (2005). New mammalian cellular systems to study mutations introduced at the break site by non-homologous end-joining. DNA Repair.

[54] Koole W., van Schendel R., Karambelas A.E., van Heteren J.T., Okihara K.L., Tijsterman M. (2014). A Polymerase Theta-dependent repair pathway suppresses extensive genomic instability at endogenous G4 DNA sites. Nat. Commun..

[55] Bosch P.C., Segura-Bayona S., Koole W., van Heteren J.T., Dewar J.M., Tijsterman M., Knipscheer P. (2014). FANCJ promotes DNA synthesis through G-quadruplex structures. EMBO J..

[56] Wu Y., Shin-ya K., Brosh R.M. (2008). FANCJ helicase defective in Fanconia anemia and breast cancer unwinds G-quadruplex DNA to defend genomic stability. Mol. Cell. Biol..

[57] Boboila C., Alt F.W., Schwer B. (2012). Classical and alternative end-joining pathways for repair of lymphocyte-specific and general DNA double-strand breaks. Adv. Immunol..

[58] Corneo B., Wendland R.L., Deriano L., Cui X., Klein I.A., Wong S.Y., Arnal S., Holub A.J., Weller G.R., Pancake B.A. (2007). Rag mutations reveal robust alternative end joining. Nature.

[59] Yan C.T., Boboila C., Souza E.K., Franco S., Hickernell T.R., Murphy M., Gumaste S., Geyer M., Zarrin A.A., Manis J.P. (2007). IgH class switching and translocations use a robust non-classical end-joining pathway. Nature.

[60] Meyer D., Fu B.X., Heyer W.D. (2015). DNA polymerases delta and lambda cooperate in repairing double-strand breaks by microhomology-mediated end-joining in Saccharomyces cerevisiae. Proc. Natl. Acad. Sci. USA.

[61] Seki M., Wood R.D. (2008). DNA polymerase theta (POLQ) can extend from mismatches and from bases opposite a (6-4) photoproduct. DNA Repair.

[62] Steitz T.A. (1999). DNA polymerases: Structural diversity and common mechanisms. J. Biol. Chem..

[63] Cannavo E., Cejka P. (2014). Sae2 promotes dsDNA endonuclease activity within Mre11-Rad50-Xrs2 to resect DNA breaks. Nature.

[64] Abdel-Monem M., Durwald H., Hoffmann-Berling H. (1976). Enzymic unwinding of DNA. 2. Chain separation by an ATP-dependent DNA unwinding enzyme. Eur. J. Biochem..

[65] Lohman T.M., Tomko E.J., Wu C.G. (2008). Non-hexameric DNA helicases and translocases: Mechanisms and regulation. Nat. Rev. Mol. Cell Biol..

[66] Singleton M.R., Dillingham M.S., Wigley D.B. (2007). Structure and mechanism of helicases and nucleic acid translocases. Annu. Rev. Biochem..

[67] Fairman-Williams M.E., Guenther U.P., Jankowsky E. (2010). SF1 and SF2 helicases: Family matters. Curr. Opin. Struct. Biol..

[68] Richards J.D., Johnson K.A., Liu H., McRobbie A.M., McMahon S., Oke M., Carter L., Naismith J.H., White M.F. (2008). Structure of the DNA repair helicase hel308 reveals DNA binding and autoinhibitory domains. J. Biol. Chem..

[69] Guy C.P., Bolt E.L. (2005). Archaeal Hel308 helicase targets replication forks in vivo and in vitro and unwinds lagging strands. Nucleic Acids Res..

[70] Pike A.C., Gomathinayagam S., Swuec P., Berti M., Zhang Y., Schnecke C., Marino F., von Delft F., Renault L., Costa A. (2015). Human RECQ1 helicase-driven DNA unwinding, annealing, and branch migration: Insights from DNA complex structures. Proc. Natl. Acad. Sci. USA.

[71] Manthei K.A., Hill M.C., Burke J.E., Butcher S.E., Keck J.L. (2015). Structural mechanisms of DNA binding and unwinding in bacterial RecQ helicases. Proc. Natl. Acad. Sci. USA.

[72] Hu Y., Raynard S., Sehorn M.G., Lu X., Bussen W., Zheng L., Stark J.M., Barnes E.L., Chi P., Janscak P. (2007). RECQL5/Recql5 helicase regulates homologous recombination and suppresses tumor formation via disruption of Rad51 presynaptic filaments. Genes Dev..

[73] Dillingham M.S., Soultanas P., Wigley D.B. (1999). Site-directed mutagenesis of motif III in PcrA helicase reveals a role in coupling ATP hydrolysis to strand separation. Nucleic Acids Res..

[74] Bugreev D.V., Yu X., Egelman E.H., Mazin A.V. (2007). Novel pro- and anti-recombination activities of the Bloom's syndrome helicase. Genes Dev..

[75] Khadka P., Croteau D.L., Bohr V.A. (2016). RECQL5 has unique strand annealing properties relative to the other human RecQ helicase proteins. DNA Repair.

[76] Yusufzai T., Kadonaga J.T. (2008). HARP is an ATP-driven annealing helicase. Science.

[77] Wu Y. (2012). Unwinding and rewinding: Double faces of helicase?. J. Nucleic Acids.

[78] Deng S.K., Gibb B., de Almeida M.J., Greene E.C., Symington L.S. (2014). RPA antagonizes microhomology-mediated repair of DNA double-strand breaks. Nat. Struct. Mol. Biol..

[79] Shrivastav M., De Haro L.P., Nickoloff J.A. (2008). Regulation of DNA double-strand break repair pathway choice. Cell Res..

[80] Branzei D., Foiani M. (2008). Regulation of DNA repair throughout the cell cycle. Nat. Rev. Mol. Cell Biol..

[81] Aparicio T., Baer R., Gautier J. (2014). DNA double-strand break repair pathway choice and cancer. DNA Repair.

[82] Moynahan M.E., Jasin M. (2010). Mitotic homologous recombination maintains genomic stability and suppresses tumorigenesis. Nat. Rev. Mol. Cell Biol..

[83] Jensen R.B., Carreira A., Kowalczykowski S.C. (2010). Purified human BRCA2 stimulates RAD51-mediated recombination. Nature.

[84] Orthwein A., Noordermeer S.M., Wilson M.D., Landry S., Enchev R.I., Sherker A., Munro M., Pinder J., Salsman J., Dellaire G. (2015). A mechanism for the suppression of homologous recombination in G1 cells. Nature.

[85] Symington L.S. (2016). Mechanism and regulation of DNA end resection in eukaryotes. Crit. Rev. Biochem. Mol. Biol..

[86] Thyme S.B., Schier A.F. (2016). Polq-Mediated End Joining Is Essential for Surviving DNA Double-Strand Breaks during Early Zebrafish Development. Cell Rep..

[87] Moldovan G.L., Dejsuphong D., Petalcorin M.I., Hofmann K., Takeda S., Boulton S.J., D'Andrea A.D. (2012). Inhibition of homologous recombination by the PCNA-interacting protein PARI. Mol. Cell.

[88] McVey M., Kaeberlein M., Tissenbaum H.A., Guarente L. (2001). The short life span of Saccharomyces cerevisiae sgs1 and srs2 mutants is a composite of normal aging processes and mitotic arrest due to defective recombination. Genetics.

[89] Veaute X., Jeusset J., Soustelle C., Kowalczykowski S.C., Le Cam E., Fabre F. (2003). The Srs2 helicase prevents recombination by disrupting Rad51 nucleoprotein filaments. Nature.

[90] Krejci L., Van Komen S., Li Y., Villemain J., Reddy M.S., Klein H., Ellenberger T., Sung P. (2003). DNA helicase Srs2 disrupts the Rad51 presynaptic filament. Nature.

[91] Ahrabi S., Sarkar S., Pfister S.X., Pirovano G., Higgins G.S., Porter A.C., Humphrey T.C. (2016). A role for human homologous recombination factors in suppressing microhomology-mediated end joining. Nucleic Acids Res..

[92] Farmer H., McCabe N., Lord C.J., Tutt A.N., Johnson D.A., Richardson T.B., Santarosa M., Dillon K.J., Hickson I., Knights C. (2005). Targeting the DNA repair defect in BRCA mutant cells as a therapeutic strategy. Nature.

[93] Bryant H.E., Schultz N., Thomas H.D., Parker K.M., Flower D., Lopez E., Kyle S., Meuth M., Curtin N.J., Helleday T. (2005). Specific killing of BRCA2-deficient tumours with inhibitors of poly(ADP-ribose) polymerase. Nature.

[94] Dale Rein I., Solberg Landsverk K., Micci F., Patzke S., Stokke T. (2015). Replication-induced DNA damage after PARP inhibition causes G2 delay, and cell line-dependent apoptosis, necrosis and multinucleation. Cell Cycle.

